# Tetra­kis[μ-2-(4-hy­droxy­phenyl)­acetato]-κ^3^ *O*,*O*′:*O*;κ^3^ *O*:*O*,*O*′-bis­{aqua­(4,4′-bi­pyridine-κ*N*)bis­[2-(4-hy­droxy­phenyl)­acetato-κ^2^ *O*,*O*]neodymium(III)} monohydrate

**DOI:** 10.1107/S1600536810043023

**Published:** 2010-10-30

**Authors:** Jia-Lu Liu, Jian-Feng Liu, Guo-Liang Zhao

**Affiliations:** a College of Chemistry and Life Sciences, Zhejiang Normal University and, Zhejiang Normal University Xingzhi College, Jinhua 321004, People’s Republic of China

## Abstract

The title complex, [Nd_2_(C_8_H_7_O_3_)_6_(C_10_H_8_N_2_)_2_(H_2_O)_2_]·H_2_O, contains two Nd atoms, six 4-hy­droxy­phenyl­acetate (hpaa) anions, two 4,4′-bipyridine mol­ecules (bipy) and two water mol­ecules; an additional water mol­ecule of solvation is also present in the crystal structure. Each of the Nd^III^ ions is nine-coordinated by seven O atoms from four hpaa ligands, an N atom from a bipy ligand and an O atom from a water mol­ecule in a distorted tricapped trigonal-prismatic geometry. The hpaa ligands are coordinated to the Nd^III^ ions in the bridging and bridging tridentate modes. Extensive O—H⋯O, O—H⋯N and C—H⋯O hydrogen bonding stabilizes the crystal structure.

## Related literature

For related structures and background literature, see: Liu *et al.* (2010 ▶); Wang *et al.* (2010 ▶); Fang & Zhang (2006 ▶); Wang & Sevov (2008 ▶).
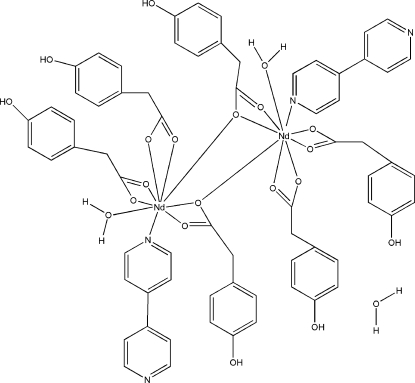


## Experimental

### 

#### Crystal data

[Nd_2_(C_8_H_7_O_3_)_6_(C_10_H_8_N_2_)_2_(H_2_O)_2_]·H_2_O
*M* *_r_* = 1561.72
Triclinic, 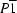

*a* = 11.77540 (1) Å
*b* = 16.3732 (2) Å
*c* = 18.4864 (2) Å
α = 83.672 (1)°
β = 71.926 (1)°
γ = 70.814 (1)°
*V* = 3199.99 (6) Å^3^
*Z* = 2
Mo *K*α radiation
μ = 1.69 mm^−1^
*T* = 296 K
0.25 × 0.24 × 0.07 mm

#### Data collection

Bruker APEXII area-detector diffractometer
Absorption correction: multi-scan (*SADABS*; Sheldrick, 1996 ▶) *T*_min_ = 0.668, *T*_max_ = 0.883
41660 measured reflections
11220 independent reflections
9780 reflections with *I* > 2σ(*I*)
*R*_int_ = 0.026

#### Refinement

*R*[*F* ^2^ > 2σ(*F* ^2^)] = 0.021
*wR*(*F* ^2^) = 0.053
*S* = 1.07
11220 reflections
875 parameters
9 restraints
H atoms treated by a mixture of independent and constrained refinement
Δρ_max_ = 0.37 e Å^−3^
Δρ_min_ = −0.51 e Å^−3^

### 

Data collection: *APEX2* (Bruker, 2006 ▶); cell refinement: *SAINT* (Bruker, 2006 ▶); data reduction: *SAINT*; program(s) used to solve structure: *SHELXS97* (Sheldrick, 2008 ▶); program(s) used to refine structure: *SHELXL97* (Sheldrick, 2008 ▶); molecular graphics: *SHELXTL* (Sheldrick, 2008 ▶); software used to prepare material for publication: *SHELXL97*.

## Supplementary Material

Crystal structure: contains datablocks I, global. DOI: 10.1107/S1600536810043023/pv2337sup1.cif

Structure factors: contains datablocks I. DOI: 10.1107/S1600536810043023/pv2337Isup2.hkl

Additional supplementary materials:  crystallographic information; 3D view; checkCIF report

## Figures and Tables

**Table 1:** Hydrogen-bond geometry (Å, °)

*D*—H⋯*A*	*D*—H	H⋯*A*	*D*⋯*A*	*D*—H⋯*A*
O3—H3*B*⋯O12^i^	0.82	1.94	2.751 (3)	170
O6—H6*B*⋯O3*W*^ii^	0.82	1.86	2.647 (3)	160
O9—H9*A*⋯O17^iii^	0.82	1.86	2.676 (3)	173
O12—H12*A*⋯O11^iv^	0.82	1.94	2.746 (2)	167
O15—H15*C*⋯O6^v^	0.82	1.91	2.725 (3)	173
O18—H18*B*⋯O9^ii^	0.82	1.95	2.763 (3)	173
O2*W*—H2*WA*⋯O5	0.82 (2)	2.02 (2)	2.772 (2)	153 (3)
O2*W*—H2*WB*⋯N2^ii^	0.81 (2)	2.04 (2)	2.840 (3)	170 (3)
O3*W*—H3*WB*⋯O3	0.82 (2)	2.02 (2)	2.811 (3)	162 (3)
O1*W*—H1*WA*⋯O13	0.81 (2)	1.98 (2)	2.764 (2)	161 (3)
O1*W*—H1*WB*⋯N4^i^	0.82 (2)	1.99 (2)	2.775 (3)	161 (3)
O3*W*—H3*WA*⋯O1^vi^	0.82 (2)	1.95 (2)	2.770 (3)	175 (4)
C6—H6*A*⋯O2^vii^	0.93	2.49	3.283 (3)	144

Symmetry codes: (i) 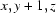; (ii) 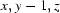; (iii) 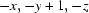; (iv) 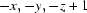; (v) 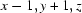; (vi) 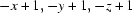; (vii) 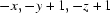.

**Fig. 1.**
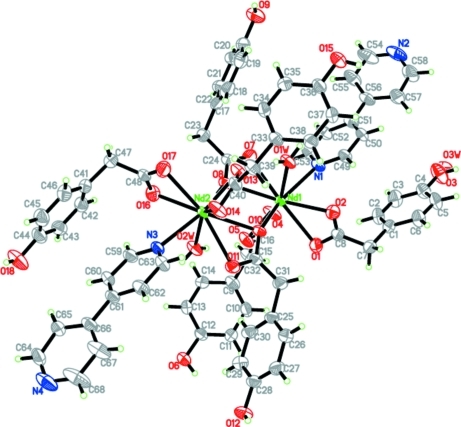
The molecular structure of the title complex, showing the atom-labelling scheme. Displacement ellipsoids are drawn at the 50% probability level.

## supplementary crystallographic information

### Comment

There has been an increasing interest in the design and synthesis of carboxylic metal-organic complexes owing to their potential practical applications, such as fluorescence, magnetism, etc. (Wang, *et al.*, 2010; Fang & Zhang, 2006; Wang & Sevov, 2008). In continuation to our research (Liu, *et al.*, 2010), we now report the preparation and crystal structure of a new neodymium^III^ complex with the ligand 4-hydroxyphenylacetic acid (hpaa).

The crystal structure of the title complex (Fig. 1) contains two Nd atoms, six (hpaa) molecules, two 4,4'-bipyridine molecules (bipy) and two water molecules; an additional water molecule of solvation is also present which is not involved in coordination. Each Nd atom is nine coordinated. The (hpaa) ligands are coordinated by two modes, bridging and bridging tridentate. The neodymium^III^ atom is in a distort capped pentagonal prism environment. The Nd—O bond lengths range from 2.4180 (15) to 2.6237 (16) Å. The Nd—N distances range from 2.6325 (19) to 2.6544 (18) Å. The Nd—O(water) bond lengths ranging from 2.4816 (16) to 2.4847 (16) Å, are slightly shorter than the other Nd—O bond distances. In addition, there are several hydrogen bonds in the crystal structure which strengthen the stability of the crystal structure (Table 1).

### Experimental

A solution of 4-hydroxyphenylacetic acid (hpaa) (30 ml) in distilled water (20 ml) was mixed with a solution of sodium hydroxide (30 mmol) in distilled water (20 ml) and the solution and diluted to 100 ml. To a 10 ml portion of this sodium salt solution a solution of Nd(NO_3_)_3_ (1 mmol) in distilled water (10 ml) was added in a dropwise fashion. After about an hour, 4,4'-bipyridine (1 mmol) in ethanol (5 ml) was slowly dripped into the solution, stirred for three hours and filtered. After allowing the filtrate to stand for a week, a mass of colorless crystals was obtained.

### Refinement

The H-atoms of the water molecules were located from differencne maps and were included in the subsequent refinement using the restraints (O—H = 0.82 (1)Å, H···H = 1.39 (2)Å) with *U*_iso_(H) = 1.5*U*_eq_(O). The remaining H atoms were positioned geometrically and refined using a riding model with O—H = 0.82 Å and C—H = 0.93 and 0.97 Å for aryl and methylene type H-atoms, respectively with *U*_iso_(H) = 1.2*U*_eq_(C) or 1.5*U*_eq_(O).

### Crystal data

**Table:**

[Nd_2_(C_8_H_7_O_3_)_6_(C_10_H_8_N_2_)_2_(H_2_O)_2_]·H_2_O	*Z* = 2
*M**_r_* = 1561.72	*F*(000) = 1576
Triclinic, *P*1	*D*_x_ = 1.621 Mg m^−^^3^
Hall symbol: -P 1	Mo *K*α radiation, λ = 0.71073 Å
*a* = 11.77540 (1) Å	Cell parameters from 9932 reflections
*b* = 16.3732 (2) Å	θ = 1.7–25.0°
*c* = 18.4864 (2) Å	µ = 1.69 mm^−^^1^
α = 83.672 (1)°	*T* = 296 K
β = 71.926 (1)°	Block, colourless
γ = 70.814 (1)°	0.25 × 0.24 × 0.07 mm
*V* = 3199.99 (6) Å^3^	

### Data collection

**Table:**

Bruker APEXII area-detector diffractometer	11220 independent reflections
Radiation source: fine-focus sealed tube	9780 reflections with *I* > 2σ(*I*)
graphite	*R*_int_ = 0.026
φ and ω scans	θ_max_ = 25.0°, θ_min_ = 1.8°
Absorption correction: multi-scan (*SADABS*; Sheldrick, 1996)	*h* = −14→14
*T*_min_ = 0.668, *T*_max_ = 0.883	*k* = −19→19
41660 measured reflections	*l* = −21→21

### Refinement

**Table:**

Refinement on *F*^2^	Primary atom site location: structure-invariant direct methods
Least-squares matrix: full	Secondary atom site location: difference Fourier map
*R*[*F*^2^ > 2σ(*F*^2^)] = 0.021	Hydrogen site location: inferred from neighbouring sites
*wR*(*F*^2^) = 0.053	H atoms treated by a mixture of independent and constrained refinement
*S* = 1.07	*w* = 1/[σ^2^(*F*_o_^2^) + (0.0242*P*)^2^ + 0.9476*P*] where *P* = (*F*_o_^2^ + 2*F*_c_^2^)/3
11220 reflections	(Δ/σ)_max_ = 0.002
875 parameters	Δρ_max_ = 0.37 e Å^−^^3^
9 restraints	Δρ_min_ = −0.51 e Å^−^^3^

### Special details

**Table:**

Geometry. All esds (except the esd in the dihedral angle between two l.s. planes) are estimated using the full covariance matrix. The cell esds are taken into account individually in the estimation of esds in distances, angles and torsion angles; correlations between esds in cell parameters are only used when they are defined by crystal symmetry. An approximate (isotropic) treatment of cell esds is used for estimating esds involving l.s. planes.
Refinement. Refinement of *F*^2^ against ALL reflections. The weighted *R*-factor *wR* and goodness of fit *S* are based on *F*^2^, conventional *R*-factors *R* are based on *F*, with *F* set to zero for negative *F*^2^. The threshold expression of *F*^2^ > σ(*F*^2^) is used only for calculating *R*-factors(gt) *etc*. and is not relevant to the choice of reflections for refinement. *R*-factors based on *F*^2^ are statistically about twice as large as those based on *F*, and *R*- factors based on ALL data will be even larger.

### Fractional atomic coordinates and isotropic or equivalent isotropic displacement parameters (Å^2^)

**Table:**

	*x*	*y*	*z*	*U*_iso_*/*U*_eq_	
Nd1	0.270706 (11)	0.364834 (7)	0.284566 (7)	0.02466 (4)	
Nd2	0.132489 (11)	0.205750 (7)	0.194233 (7)	0.02465 (4)	
N1	0.3145 (2)	0.51275 (12)	0.28312 (12)	0.0344 (5)	
N2	0.3992 (3)	0.92555 (16)	0.2132 (2)	0.0641 (8)	
N3	0.08245 (19)	0.06055 (12)	0.18750 (11)	0.0321 (5)	
N4	0.0249 (3)	−0.36120 (16)	0.2350 (2)	0.0665 (8)	
C1	0.2239 (2)	0.51251 (16)	0.51879 (13)	0.0336 (6)	
C2	0.3397 (3)	0.51654 (18)	0.47391 (15)	0.0447 (7)	
H2A	0.3955	0.4690	0.4448	0.054*	
C3	0.3749 (3)	0.58985 (19)	0.47123 (16)	0.0498 (7)	
H3A	0.4529	0.5917	0.4399	0.060*	
C4	0.2944 (3)	0.65967 (17)	0.51496 (16)	0.0431 (7)	
C5	0.1802 (3)	0.65599 (18)	0.56189 (16)	0.0467 (7)	
H5A	0.1267	0.7025	0.5930	0.056*	
C6	0.1446 (3)	0.58383 (17)	0.56302 (15)	0.0434 (7)	
H6A	0.0659	0.5828	0.5940	0.052*	
C7	0.1849 (3)	0.43233 (17)	0.52304 (14)	0.0400 (6)	
H7A	0.2317	0.3874	0.5511	0.048*	
H7B	0.0963	0.4455	0.5504	0.048*	
C8	0.2082 (2)	0.39955 (16)	0.44499 (14)	0.0339 (6)	
C9	0.6231 (2)	0.08712 (16)	0.30460 (15)	0.0368 (6)	
C10	0.6052 (3)	0.05562 (17)	0.37775 (16)	0.0461 (7)	
H10A	0.5935	0.0919	0.4165	0.055*	
C11	0.6039 (3)	−0.02832 (17)	0.39577 (15)	0.0466 (7)	
H11A	0.5932	−0.0482	0.4458	0.056*	
C12	0.6186 (2)	−0.08206 (15)	0.33954 (14)	0.0369 (6)	
C13	0.6359 (3)	−0.05165 (17)	0.26549 (14)	0.0422 (7)	
H13A	0.6463	−0.0879	0.2270	0.051*	
C14	0.6378 (3)	0.03113 (16)	0.24858 (15)	0.0408 (6)	
H14A	0.6493	0.0506	0.1984	0.049*	
C15	0.6270 (3)	0.17779 (16)	0.28492 (18)	0.0471 (7)	
H15A	0.6946	0.1763	0.2384	0.057*	
H15B	0.6472	0.1986	0.3250	0.057*	
C16	0.5074 (2)	0.24161 (15)	0.27414 (14)	0.0330 (6)	
C17	0.2717 (3)	0.45494 (17)	0.01296 (14)	0.0415 (7)	
C18	0.1461 (3)	0.50163 (19)	0.02788 (14)	0.0482 (7)	
H18A	0.0863	0.4747	0.0534	0.058*	
C19	0.1064 (3)	0.58758 (19)	0.00594 (15)	0.0450 (7)	
H19A	0.0210	0.6179	0.0170	0.054*	
C20	0.1948 (3)	0.62755 (17)	−0.03244 (14)	0.0387 (6)	
C21	0.3209 (3)	0.58215 (17)	−0.04733 (15)	0.0419 (6)	
H21A	0.3809	0.6091	−0.0724	0.050*	
C22	0.3581 (3)	0.49691 (17)	−0.02502 (15)	0.0425 (7)	
H22A	0.4434	0.4668	−0.0358	0.051*	
C23	0.3127 (3)	0.36227 (18)	0.03879 (15)	0.0576 (9)	
H23A	0.3977	0.3338	0.0079	0.069*	
H23B	0.2583	0.3328	0.0310	0.069*	
C24	0.3091 (2)	0.35461 (15)	0.12153 (13)	0.0327 (6)	
C25	0.1422 (3)	0.11229 (16)	0.47574 (14)	0.0380 (6)	
C26	0.0397 (3)	0.10565 (17)	0.53565 (14)	0.0422 (6)	
H26A	−0.0291	0.1547	0.5516	0.051*	
C27	0.0379 (3)	0.02725 (17)	0.57209 (14)	0.0413 (6)	
H27A	−0.0311	0.0239	0.6125	0.050*	
C28	0.1390 (2)	−0.04569 (16)	0.54792 (14)	0.0349 (6)	
C29	0.2418 (3)	−0.04044 (18)	0.48871 (15)	0.0414 (6)	
H29A	0.3102	−0.0896	0.4724	0.050*	
C30	0.2424 (3)	0.03838 (18)	0.45375 (15)	0.0419 (6)	
H30A	0.3125	0.0417	0.4142	0.050*	
C31	0.1436 (3)	0.19656 (17)	0.43415 (14)	0.0458 (7)	
H31A	0.2246	0.2043	0.4272	0.055*	
H31B	0.0800	0.2434	0.4658	0.055*	
C32	0.1205 (2)	0.20377 (15)	0.35757 (13)	0.0291 (5)	
C33	−0.2574 (2)	0.48110 (15)	0.24444 (14)	0.0308 (5)	
C34	−0.2146 (2)	0.51905 (16)	0.17483 (14)	0.0369 (6)	
H34A	−0.1765	0.4848	0.1316	0.044*	
C35	−0.2266 (3)	0.60524 (16)	0.16765 (14)	0.0400 (6)	
H35A	−0.1973	0.6287	0.1200	0.048*	
C36	−0.2821 (2)	0.65779 (16)	0.23092 (14)	0.0367 (6)	
C37	−0.3248 (3)	0.62160 (16)	0.30103 (14)	0.0400 (6)	
H37A	−0.3622	0.6560	0.3441	0.048*	
C38	−0.3121 (2)	0.53431 (16)	0.30741 (14)	0.0373 (6)	
H38A	−0.3408	0.5108	0.3551	0.045*	
C39	−0.2452 (2)	0.38659 (15)	0.25102 (15)	0.0375 (6)	
H39A	−0.2904	0.3748	0.3026	0.045*	
H39B	−0.2863	0.3744	0.2172	0.045*	
C40	−0.1139 (2)	0.32540 (15)	0.23301 (13)	0.0303 (5)	
C41	0.2356 (3)	0.05071 (16)	−0.04968 (13)	0.0381 (6)	
C42	0.3543 (3)	−0.00084 (17)	−0.04648 (14)	0.0401 (6)	
H42A	0.4115	0.0256	−0.0433	0.048*	
C43	0.3896 (3)	−0.08951 (17)	−0.04784 (14)	0.0433 (6)	
H43A	0.4698	−0.1224	−0.0460	0.052*	
C44	0.3055 (3)	−0.12956 (18)	−0.05194 (16)	0.0470 (7)	
C45	0.1880 (3)	−0.08015 (19)	−0.05647 (16)	0.0493 (7)	
H45A	0.1317	−0.1069	−0.0603	0.059*	
C46	0.1535 (3)	0.00887 (18)	−0.05533 (14)	0.0438 (7)	
H46A	0.0739	0.0415	−0.0584	0.053*	
C47	0.1944 (3)	0.14797 (17)	−0.04442 (14)	0.0449 (7)	
H47A	0.1165	0.1723	−0.0578	0.054*	
H47B	0.2576	0.1696	−0.0808	0.054*	
C48	0.1751 (3)	0.17737 (15)	0.03463 (14)	0.0350 (6)	
C49	0.2327 (2)	0.58181 (15)	0.32258 (14)	0.0364 (6)	
H49A	0.1603	0.5758	0.3583	0.044*	
C50	0.2504 (3)	0.66216 (15)	0.31305 (14)	0.0381 (6)	
H50A	0.1908	0.7087	0.3420	0.046*	
C51	0.3571 (2)	0.67304 (15)	0.26029 (15)	0.0356 (6)	
C52	0.4435 (2)	0.60051 (16)	0.22073 (16)	0.0430 (7)	
H52A	0.5176	0.6043	0.1854	0.052*	
C53	0.4191 (3)	0.52275 (16)	0.23395 (16)	0.0415 (6)	
H53A	0.4786	0.4747	0.2072	0.050*	
C54	0.4499 (3)	0.8642 (2)	0.1607 (2)	0.0668 (10)	
H54A	0.4940	0.8778	0.1122	0.080*	
C55	0.4411 (3)	0.78141 (18)	0.17384 (19)	0.0564 (8)	
H55A	0.4787	0.7410	0.1350	0.068*	
C56	0.3761 (3)	0.75937 (16)	0.24497 (17)	0.0420 (7)	
C57	0.3254 (3)	0.82212 (18)	0.30020 (18)	0.0592 (9)	
H57A	0.2826	0.8101	0.3495	0.071*	
C58	0.3392 (4)	0.9033 (2)	0.2810 (2)	0.0715 (10)	
H58A	0.3035	0.9449	0.3189	0.086*	
C59	0.1674 (3)	−0.00571 (16)	0.14551 (14)	0.0382 (6)	
H59A	0.2399	0.0029	0.1120	0.046*	
C60	0.1531 (3)	−0.08665 (16)	0.14932 (15)	0.0407 (6)	
H60A	0.2148	−0.1305	0.1181	0.049*	
C61	0.0480 (2)	−0.10279 (15)	0.19916 (14)	0.0351 (6)	
C62	−0.0423 (2)	−0.03258 (16)	0.24109 (15)	0.0396 (6)	
H62A	−0.1164	−0.0391	0.2744	0.047*	
C63	−0.0223 (2)	0.04660 (16)	0.23338 (15)	0.0370 (6)	
H63A	−0.0848	0.0927	0.2615	0.044*	
C64	0.1031 (4)	−0.33921 (19)	0.1736 (2)	0.0719 (11)	
H64A	0.1558	−0.3822	0.1385	0.086*	
C65	0.1115 (4)	−0.25666 (18)	0.15800 (17)	0.0617 (9)	
H65A	0.1674	−0.2451	0.1132	0.074*	
C66	0.0362 (3)	−0.19106 (16)	0.20950 (16)	0.0417 (7)	
C67	−0.0466 (3)	−0.21345 (19)	0.2739 (2)	0.0596 (9)	
H67A	−0.0998	−0.1721	0.3104	0.072*	
C68	−0.0495 (3)	−0.2978 (2)	0.2833 (3)	0.0740 (11)	
H68A	−0.1074	−0.3109	0.3263	0.089*	
O1W	0.07774 (17)	0.46117 (11)	0.25536 (11)	0.0393 (4)	
H1WA	0.034 (2)	0.4394 (16)	0.2422 (16)	0.059*	
H1WB	0.045 (3)	0.5134 (10)	0.2533 (17)	0.059*	
O1	0.30329 (18)	0.33594 (11)	0.41748 (10)	0.0445 (5)	
O2W	0.33238 (17)	0.10872 (11)	0.21403 (12)	0.0406 (4)	
H2WA	0.379 (2)	0.1269 (16)	0.2276 (17)	0.061*	
H2WB	0.360 (3)	0.0565 (11)	0.2138 (17)	0.061*	
O2	0.13434 (16)	0.43730 (11)	0.40659 (9)	0.0384 (4)	
O3	0.3315 (2)	0.73205 (14)	0.51087 (13)	0.0660 (6)	
H3B	0.2736	0.7706	0.5367	0.057 (10)*	
O3W	0.5579 (2)	0.76681 (14)	0.49067 (13)	0.0566 (5)	
H3WA	0.603 (3)	0.736 (2)	0.5157 (18)	0.085*	
H3WB	0.502 (2)	0.746 (2)	0.495 (2)	0.085*	
O4	0.49884 (16)	0.32022 (11)	0.26571 (11)	0.0469 (5)	
O5	0.41618 (16)	0.21614 (10)	0.27630 (10)	0.0398 (4)	
O6	0.6197 (2)	−0.16628 (11)	0.35255 (11)	0.0572 (6)	
H6B	0.5929	−0.1745	0.3984	0.086*	
O7	0.35297 (18)	0.40041 (12)	0.14693 (10)	0.0463 (5)	
O8	0.25908 (15)	0.30193 (10)	0.16424 (9)	0.0306 (4)	
O9	0.16217 (19)	0.71234 (12)	−0.05605 (12)	0.0557 (5)	
H9A	0.0916	0.7266	−0.0613	0.084*	
O10	0.14216 (15)	0.26635 (10)	0.31474 (9)	0.0294 (4)	
O11	0.08199 (16)	0.15084 (10)	0.33586 (9)	0.0370 (4)	
O12	0.14175 (18)	−0.12543 (11)	0.58121 (10)	0.0462 (5)	
H12A	0.0698	−0.1254	0.6038	0.069*	
O13	−0.01952 (15)	0.35251 (10)	0.20624 (10)	0.0381 (4)	
O14	−0.09706 (16)	0.24565 (10)	0.24352 (11)	0.0426 (4)	
O15	−0.2924 (2)	0.74313 (11)	0.22082 (11)	0.0532 (5)	
H15C	−0.3189	0.7666	0.2624	0.080*	
O16	0.26326 (17)	0.15156 (11)	0.06384 (10)	0.0407 (4)	
O17	0.06975 (18)	0.22670 (12)	0.07132 (10)	0.0480 (5)	
O18	0.3439 (2)	−0.21795 (13)	−0.05134 (14)	0.0743 (7)	
H18B	0.2876	−0.2349	−0.0548	0.111*	

### Atomic displacement parameters (Å^2^)

**Table:**

	*U*^11^	*U*^22^	*U*^33^	*U*^12^	*U*^13^	*U*^23^
Nd1	0.02896 (8)	0.01707 (7)	0.03149 (7)	−0.00971 (5)	−0.01188 (6)	0.00237 (5)
Nd2	0.02733 (8)	0.01603 (7)	0.03255 (8)	−0.00763 (5)	−0.01066 (6)	0.00014 (5)
N1	0.0364 (13)	0.0231 (11)	0.0473 (13)	−0.0117 (10)	−0.0151 (10)	0.0010 (9)
N2	0.0659 (19)	0.0310 (14)	0.113 (2)	−0.0255 (14)	−0.0456 (18)	0.0175 (16)
N3	0.0381 (13)	0.0221 (10)	0.0388 (12)	−0.0111 (9)	−0.0133 (10)	0.0000 (9)
N4	0.084 (2)	0.0321 (15)	0.115 (3)	−0.0280 (15)	−0.069 (2)	0.0194 (16)
C1	0.0357 (15)	0.0368 (14)	0.0301 (13)	−0.0119 (12)	−0.0107 (11)	−0.0017 (11)
C2	0.0404 (17)	0.0411 (16)	0.0478 (16)	−0.0082 (13)	−0.0062 (13)	−0.0148 (13)
C3	0.0367 (16)	0.0538 (18)	0.0545 (18)	−0.0175 (14)	−0.0003 (14)	−0.0099 (14)
C4	0.0382 (16)	0.0398 (16)	0.0551 (17)	−0.0167 (13)	−0.0131 (14)	−0.0027 (13)
C5	0.0406 (17)	0.0355 (15)	0.0563 (18)	−0.0074 (13)	−0.0033 (14)	−0.0150 (13)
C6	0.0336 (15)	0.0449 (16)	0.0494 (16)	−0.0149 (13)	−0.0026 (13)	−0.0106 (13)
C7	0.0505 (17)	0.0384 (15)	0.0335 (14)	−0.0184 (13)	−0.0109 (12)	0.0001 (11)
C8	0.0436 (16)	0.0290 (14)	0.0335 (13)	−0.0201 (13)	−0.0088 (12)	0.0022 (11)
C9	0.0255 (14)	0.0283 (13)	0.0579 (17)	−0.0037 (11)	−0.0188 (12)	−0.0004 (12)
C10	0.0608 (19)	0.0304 (15)	0.0484 (17)	−0.0046 (13)	−0.0250 (15)	−0.0096 (12)
C11	0.066 (2)	0.0367 (16)	0.0339 (14)	−0.0104 (14)	−0.0165 (14)	−0.0007 (12)
C12	0.0429 (16)	0.0258 (13)	0.0423 (15)	−0.0101 (12)	−0.0140 (12)	0.0015 (11)
C13	0.0577 (19)	0.0342 (15)	0.0361 (14)	−0.0133 (13)	−0.0146 (13)	−0.0068 (11)
C14	0.0483 (17)	0.0340 (15)	0.0379 (15)	−0.0109 (13)	−0.0131 (13)	0.0040 (12)
C15	0.0371 (16)	0.0281 (14)	0.082 (2)	−0.0089 (12)	−0.0286 (15)	0.0059 (14)
C16	0.0327 (14)	0.0267 (13)	0.0400 (14)	−0.0078 (11)	−0.0122 (11)	−0.0021 (11)
C17	0.069 (2)	0.0403 (15)	0.0274 (13)	−0.0289 (15)	−0.0194 (13)	0.0045 (11)
C18	0.068 (2)	0.060 (2)	0.0315 (14)	−0.0443 (18)	−0.0107 (14)	0.0042 (13)
C19	0.0449 (17)	0.0550 (18)	0.0403 (15)	−0.0203 (15)	−0.0125 (13)	−0.0073 (13)
C20	0.0484 (17)	0.0359 (15)	0.0420 (15)	−0.0182 (13)	−0.0231 (13)	0.0027 (12)
C21	0.0488 (18)	0.0377 (15)	0.0485 (16)	−0.0251 (14)	−0.0182 (13)	0.0097 (12)
C22	0.0488 (17)	0.0375 (15)	0.0472 (16)	−0.0164 (14)	−0.0210 (14)	0.0058 (12)
C23	0.111 (3)	0.0376 (16)	0.0385 (16)	−0.0399 (18)	−0.0268 (17)	0.0067 (13)
C24	0.0428 (15)	0.0255 (13)	0.0308 (13)	−0.0129 (12)	−0.0104 (12)	0.0024 (10)
C25	0.0587 (18)	0.0351 (15)	0.0343 (14)	−0.0269 (14)	−0.0236 (13)	0.0098 (11)
C26	0.0539 (18)	0.0334 (14)	0.0403 (15)	−0.0115 (13)	−0.0182 (14)	0.0026 (12)
C27	0.0473 (17)	0.0431 (16)	0.0347 (14)	−0.0202 (14)	−0.0093 (13)	0.0060 (12)
C28	0.0434 (16)	0.0323 (14)	0.0364 (14)	−0.0189 (13)	−0.0170 (12)	0.0080 (11)
C29	0.0411 (16)	0.0428 (16)	0.0416 (15)	−0.0149 (13)	−0.0138 (13)	0.0053 (12)
C30	0.0469 (17)	0.0485 (17)	0.0367 (15)	−0.0269 (15)	−0.0117 (13)	0.0101 (12)
C31	0.075 (2)	0.0388 (16)	0.0403 (15)	−0.0341 (15)	−0.0263 (15)	0.0096 (12)
C32	0.0314 (14)	0.0226 (12)	0.0337 (13)	−0.0107 (11)	−0.0085 (11)	0.0025 (10)
C33	0.0236 (13)	0.0245 (12)	0.0432 (14)	−0.0033 (10)	−0.0118 (11)	−0.0026 (11)
C34	0.0395 (15)	0.0308 (14)	0.0366 (14)	−0.0051 (12)	−0.0091 (12)	−0.0086 (11)
C35	0.0500 (17)	0.0358 (15)	0.0316 (14)	−0.0139 (13)	−0.0089 (12)	0.0027 (11)
C36	0.0415 (16)	0.0270 (13)	0.0443 (15)	−0.0098 (12)	−0.0169 (13)	−0.0017 (11)
C37	0.0493 (17)	0.0309 (14)	0.0358 (14)	−0.0048 (13)	−0.0122 (13)	−0.0091 (11)
C38	0.0384 (15)	0.0326 (14)	0.0348 (14)	−0.0063 (12)	−0.0080 (12)	0.0010 (11)
C39	0.0292 (14)	0.0273 (13)	0.0533 (16)	−0.0059 (11)	−0.0107 (12)	−0.0024 (12)
C40	0.0320 (14)	0.0223 (13)	0.0393 (14)	−0.0078 (11)	−0.0141 (11)	−0.0027 (10)
C41	0.0501 (17)	0.0341 (14)	0.0290 (13)	−0.0130 (13)	−0.0092 (12)	−0.0036 (11)
C42	0.0450 (17)	0.0414 (16)	0.0382 (15)	−0.0220 (14)	−0.0086 (12)	−0.0016 (12)
C43	0.0438 (17)	0.0376 (16)	0.0446 (16)	−0.0101 (13)	−0.0108 (13)	0.0013 (12)
C44	0.056 (2)	0.0351 (16)	0.0508 (17)	−0.0201 (14)	−0.0102 (14)	−0.0015 (13)
C45	0.055 (2)	0.0476 (18)	0.0532 (18)	−0.0263 (16)	−0.0138 (15)	−0.0044 (14)
C46	0.0430 (17)	0.0467 (17)	0.0403 (15)	−0.0116 (14)	−0.0102 (13)	−0.0076 (12)
C47	0.0617 (19)	0.0361 (15)	0.0345 (14)	−0.0120 (14)	−0.0143 (14)	0.0001 (12)
C48	0.0498 (17)	0.0210 (13)	0.0355 (14)	−0.0146 (12)	−0.0114 (13)	0.0019 (11)
C49	0.0390 (15)	0.0280 (14)	0.0419 (15)	−0.0118 (12)	−0.0104 (12)	0.0011 (11)
C50	0.0468 (17)	0.0218 (13)	0.0450 (15)	−0.0084 (12)	−0.0142 (13)	−0.0029 (11)
C51	0.0394 (15)	0.0223 (13)	0.0528 (16)	−0.0132 (12)	−0.0222 (13)	0.0057 (11)
C52	0.0292 (15)	0.0298 (14)	0.0679 (19)	−0.0121 (12)	−0.0095 (13)	0.0031 (13)
C53	0.0362 (16)	0.0236 (13)	0.0624 (18)	−0.0101 (12)	−0.0089 (14)	−0.0048 (12)
C54	0.052 (2)	0.0389 (19)	0.104 (3)	−0.0221 (16)	−0.0156 (19)	0.0216 (19)
C55	0.0459 (18)	0.0321 (16)	0.082 (2)	−0.0153 (14)	−0.0042 (16)	0.0023 (15)
C56	0.0418 (16)	0.0254 (14)	0.0667 (19)	−0.0128 (12)	−0.0265 (15)	0.0052 (13)
C57	0.093 (3)	0.0367 (17)	0.062 (2)	−0.0328 (17)	−0.0308 (19)	0.0038 (14)
C58	0.108 (3)	0.0358 (18)	0.093 (3)	−0.032 (2)	−0.049 (2)	−0.0025 (18)
C59	0.0447 (16)	0.0278 (14)	0.0405 (15)	−0.0140 (12)	−0.0083 (13)	0.0024 (11)
C60	0.0547 (18)	0.0236 (13)	0.0418 (15)	−0.0085 (13)	−0.0142 (13)	−0.0035 (11)
C61	0.0463 (16)	0.0247 (13)	0.0460 (15)	−0.0146 (12)	−0.0280 (13)	0.0060 (11)
C62	0.0365 (15)	0.0310 (14)	0.0545 (17)	−0.0160 (12)	−0.0136 (13)	0.0039 (12)
C63	0.0364 (15)	0.0247 (13)	0.0501 (16)	−0.0091 (12)	−0.0118 (13)	−0.0048 (11)
C64	0.136 (4)	0.0330 (17)	0.073 (2)	−0.028 (2)	−0.066 (3)	0.0020 (16)
C65	0.116 (3)	0.0343 (17)	0.0529 (18)	−0.0323 (18)	−0.0421 (19)	0.0052 (14)
C66	0.0543 (18)	0.0262 (14)	0.0615 (18)	−0.0180 (13)	−0.0373 (15)	0.0084 (13)
C67	0.0435 (18)	0.0326 (16)	0.103 (3)	−0.0135 (14)	−0.0239 (18)	0.0126 (16)
C68	0.050 (2)	0.0388 (19)	0.141 (4)	−0.0242 (17)	−0.038 (2)	0.027 (2)
O1W	0.0440 (12)	0.0192 (9)	0.0619 (12)	−0.0072 (8)	−0.0286 (10)	0.0002 (9)
O1	0.0541 (13)	0.0363 (11)	0.0409 (10)	−0.0022 (9)	−0.0219 (9)	−0.0056 (8)
O2W	0.0381 (11)	0.0217 (9)	0.0681 (13)	−0.0072 (8)	−0.0264 (9)	0.0000 (9)
O2	0.0369 (10)	0.0406 (10)	0.0386 (10)	−0.0120 (9)	−0.0122 (8)	−0.0012 (8)
O3	0.0504 (14)	0.0431 (12)	0.1041 (18)	−0.0248 (11)	−0.0063 (13)	−0.0148 (13)
O3W	0.0596 (15)	0.0539 (14)	0.0622 (14)	−0.0233 (12)	−0.0267 (12)	0.0194 (10)
O4	0.0361 (11)	0.0234 (10)	0.0840 (14)	−0.0116 (8)	−0.0223 (10)	0.0096 (9)
O5	0.0338 (10)	0.0245 (9)	0.0676 (12)	−0.0076 (8)	−0.0240 (9)	−0.0043 (8)
O6	0.0937 (17)	0.0321 (11)	0.0460 (11)	−0.0266 (11)	−0.0135 (11)	0.0007 (9)
O7	0.0681 (13)	0.0516 (12)	0.0371 (10)	−0.0444 (11)	−0.0149 (9)	0.0063 (8)
O8	0.0360 (10)	0.0230 (8)	0.0345 (9)	−0.0143 (8)	−0.0084 (8)	0.0035 (7)
O9	0.0591 (14)	0.0370 (11)	0.0821 (15)	−0.0156 (10)	−0.0386 (12)	0.0092 (10)
O10	0.0338 (10)	0.0220 (8)	0.0348 (9)	−0.0132 (7)	−0.0099 (7)	0.0045 (7)
O11	0.0513 (11)	0.0341 (10)	0.0368 (9)	−0.0265 (9)	−0.0169 (8)	0.0075 (8)
O12	0.0506 (12)	0.0342 (10)	0.0549 (12)	−0.0208 (9)	−0.0137 (9)	0.0130 (9)
O13	0.0274 (10)	0.0226 (9)	0.0656 (12)	−0.0073 (8)	−0.0146 (9)	−0.0041 (8)
O14	0.0317 (10)	0.0221 (9)	0.0688 (13)	−0.0078 (8)	−0.0090 (9)	0.0015 (8)
O15	0.0797 (15)	0.0260 (10)	0.0565 (12)	−0.0175 (10)	−0.0229 (11)	0.0009 (9)
O16	0.0387 (11)	0.0405 (11)	0.0439 (10)	−0.0121 (9)	−0.0120 (9)	−0.0053 (8)
O17	0.0501 (13)	0.0438 (11)	0.0433 (11)	0.0028 (10)	−0.0203 (10)	−0.0081 (9)
O18	0.0785 (17)	0.0333 (12)	0.116 (2)	−0.0211 (12)	−0.0315 (15)	−0.0007 (12)

### Geometric parameters (Å, °)

**Table:**

Nd1—O4	2.4611 (17)	C28—O12	1.376 (3)
Nd1—O5	2.4624 (16)	C28—C29	1.377 (4)
Nd1—O10	2.4666 (15)	C29—C30	1.379 (4)
Nd1—O1W	2.4820 (16)	C29—H29A	0.9300
Nd1—O2	2.4829 (17)	C30—H30A	0.9300
Nd1—O7	2.5033 (17)	C31—C32	1.508 (3)
Nd1—O1	2.5724 (16)	C31—H31A	0.9700
Nd1—O8	2.6104 (15)	C31—H31B	0.9700
Nd1—N1	2.6326 (19)	C32—O11	1.251 (3)
Nd1—C16	2.816 (2)	C32—O10	1.269 (3)
Nd1—C8	2.899 (2)	C33—C38	1.385 (3)
Nd1—C24	2.921 (2)	C33—C34	1.387 (3)
Nd2—O8	2.4179 (15)	C33—C39	1.501 (3)
Nd2—O14	2.4506 (17)	C34—C35	1.367 (3)
Nd2—O13	2.4640 (16)	C34—H34A	0.9300
Nd2—O2W	2.4793 (16)	C35—C36	1.383 (3)
Nd2—O16	2.4978 (17)	C35—H35A	0.9300
Nd2—O17	2.5552 (17)	C36—O15	1.359 (3)
Nd2—O10	2.5845 (15)	C36—C37	1.381 (4)
Nd2—O11	2.6236 (16)	C37—C38	1.383 (3)
Nd2—N3	2.6545 (18)	C37—H37A	0.9300
Nd2—C40	2.838 (2)	C38—H38A	0.9300
Nd2—C48	2.899 (2)	C39—C40	1.498 (3)
Nd2—C32	2.976 (2)	C39—H39A	0.9700
N1—C49	1.330 (3)	C39—H39B	0.9700
N1—C53	1.332 (3)	C40—O14	1.257 (3)
N2—C58	1.313 (4)	C40—O13	1.269 (3)
N2—C54	1.329 (4)	C41—C46	1.388 (4)
N3—C59	1.330 (3)	C41—C42	1.391 (4)
N3—C63	1.337 (3)	C41—C47	1.509 (3)
N4—C64	1.322 (5)	C42—C43	1.373 (4)
N4—C68	1.328 (5)	C42—H42A	0.9300
C1—C2	1.378 (4)	C43—C44	1.379 (4)
C1—C6	1.387 (3)	C43—H43A	0.9300
C1—C7	1.513 (3)	C44—O18	1.367 (3)
C2—C3	1.384 (4)	C44—C45	1.377 (4)
C2—H2A	0.9300	C45—C46	1.379 (4)
C3—C4	1.369 (4)	C45—H45A	0.9300
C3—H3A	0.9300	C46—H46A	0.9300
C4—C5	1.372 (4)	C47—C48	1.515 (3)
C4—O3	1.379 (3)	C47—H47A	0.9700
C5—C6	1.374 (4)	C47—H47B	0.9700
C5—H5A	0.9300	C48—O16	1.248 (3)
C6—H6A	0.9300	C48—O17	1.262 (3)
C7—C8	1.508 (3)	C49—C50	1.383 (3)
C7—H7A	0.9700	C49—H49A	0.9300
C7—H7B	0.9700	C50—C51	1.382 (4)
C8—O2	1.255 (3)	C50—H50A	0.9300
C8—O1	1.262 (3)	C51—C52	1.384 (4)
C9—C10	1.371 (4)	C51—C56	1.486 (3)
C9—C14	1.392 (3)	C52—C53	1.376 (3)
C9—C15	1.501 (3)	C52—H52A	0.9300
C10—C11	1.382 (4)	C53—H53A	0.9300
C10—H10A	0.9300	C54—C55	1.383 (4)
C11—C12	1.369 (3)	C54—H54A	0.9300
C11—H11A	0.9300	C55—C56	1.378 (4)
C12—O6	1.370 (3)	C55—H55A	0.9300
C12—C13	1.381 (3)	C56—C57	1.380 (4)
C13—C14	1.363 (3)	C57—C58	1.386 (4)
C13—H13A	0.9300	C57—H57A	0.9300
C14—H14A	0.9300	C58—H58A	0.9300
C15—C16	1.509 (3)	C59—C60	1.381 (3)
C15—H15A	0.9700	C59—H59A	0.9300
C15—H15B	0.9700	C60—C61	1.380 (4)
C16—O4	1.254 (3)	C60—H60A	0.9300
C16—O5	1.262 (3)	C61—C62	1.391 (4)
C17—C18	1.377 (4)	C61—C66	1.483 (3)
C17—C22	1.382 (4)	C62—C63	1.377 (3)
C17—C23	1.505 (4)	C62—H62A	0.9300
C18—C19	1.387 (4)	C63—H63A	0.9300
C18—H18A	0.9300	C64—C65	1.380 (4)
C19—C20	1.380 (4)	C64—H64A	0.9300
C19—H19A	0.9300	C65—C66	1.386 (4)
C20—O9	1.373 (3)	C65—H65A	0.9300
C20—C21	1.378 (4)	C66—C67	1.386 (4)
C21—C22	1.376 (3)	C67—C68	1.383 (4)
C21—H21A	0.9300	C67—H67A	0.9300
C22—H22A	0.9300	C68—H68A	0.9300
C23—C24	1.509 (3)	O1W—H1WA	0.81 (2)
C23—H23A	0.9700	O1W—H1WB	0.82 (2)
C23—H23B	0.9700	O2W—H2WA	0.82 (2)
C24—O7	1.238 (3)	O2W—H2WB	0.81 (2)
C24—O8	1.269 (3)	O3—H3B	0.8200
C25—C30	1.376 (4)	O3W—H3WA	0.82 (2)
C25—C26	1.388 (4)	O3W—H3WB	0.82 (2)
C25—C31	1.506 (3)	O6—H6B	0.8200
C26—C27	1.386 (3)	O9—H9A	0.8200
C26—H26A	0.9300	O12—H12A	0.8200
C27—C28	1.377 (4)	O15—H15C	0.8200
C27—H27A	0.9300	O18—H18B	0.8200
			
O4—Nd1—O5	52.77 (5)	C19—C18—H18A	119.0
O4—Nd1—O10	125.18 (5)	C20—C19—C18	119.2 (3)
O5—Nd1—O10	72.80 (5)	C20—C19—H19A	120.4
O4—Nd1—O1W	151.17 (6)	C18—C19—H19A	120.4
O5—Nd1—O1W	144.97 (5)	O9—C20—C21	117.9 (2)
O10—Nd1—O1W	79.96 (5)	O9—C20—C19	122.4 (3)
O4—Nd1—O2	116.30 (6)	C21—C20—C19	119.7 (2)
O5—Nd1—O2	123.58 (6)	C22—C21—C20	119.9 (3)
O10—Nd1—O2	86.53 (5)	C22—C21—H21A	120.0
O1W—Nd1—O2	75.10 (6)	C20—C21—H21A	120.0
O4—Nd1—O7	77.79 (6)	C21—C22—C17	121.6 (3)
O5—Nd1—O7	95.05 (6)	C21—C22—H22A	119.2
O10—Nd1—O7	116.73 (5)	C17—C22—H22A	119.2
O1W—Nd1—O7	77.80 (7)	C17—C23—C24	112.3 (2)
O2—Nd1—O7	140.21 (6)	C17—C23—H23A	109.1
O4—Nd1—O1	73.13 (6)	C24—C23—H23A	109.1
O5—Nd1—O1	76.34 (6)	C17—C23—H23B	109.1
O10—Nd1—O1	89.72 (5)	C24—C23—H23B	109.1
O1W—Nd1—O1	125.79 (6)	H23A—C23—H23B	107.9
O2—Nd1—O1	51.08 (6)	O7—C24—O8	121.1 (2)
O7—Nd1—O1	148.77 (6)	O7—C24—C23	119.8 (2)
O4—Nd1—O8	102.01 (6)	O8—C24—C23	119.1 (2)
O5—Nd1—O8	75.76 (5)	O7—C24—Nd1	58.32 (12)
O10—Nd1—O8	66.52 (5)	O8—C24—Nd1	63.32 (12)
O1W—Nd1—O8	73.40 (5)	C23—C24—Nd1	171.22 (19)
O2—Nd1—O8	141.38 (5)	C30—C25—C26	117.8 (2)
O7—Nd1—O8	50.48 (5)	C30—C25—C31	120.5 (3)
O1—Nd1—O8	147.63 (5)	C26—C25—C31	121.7 (3)
O4—Nd1—N1	76.87 (6)	C27—C26—C25	121.2 (3)
O5—Nd1—N1	129.58 (6)	C27—C26—H26A	119.4
O10—Nd1—N1	155.81 (6)	C25—C26—H26A	119.4
O1W—Nd1—N1	82.04 (6)	C28—C27—C26	119.5 (2)
O2—Nd1—N1	73.28 (6)	C28—C27—H27A	120.3
O7—Nd1—N1	74.58 (6)	C26—C27—H27A	120.3
O1—Nd1—N1	87.81 (6)	O12—C28—C29	117.8 (2)
O8—Nd1—N1	122.92 (5)	O12—C28—C27	122.0 (2)
O4—Nd1—C16	26.40 (6)	C29—C28—C27	120.2 (2)
O5—Nd1—C16	26.59 (6)	C28—C29—C30	119.4 (3)
O10—Nd1—C16	98.80 (6)	C28—C29—H29A	120.3
O1W—Nd1—C16	163.55 (7)	C30—C29—H29A	120.3
O2—Nd1—C16	121.30 (6)	C25—C30—C29	121.9 (3)
O7—Nd1—C16	88.36 (7)	C25—C30—H30A	119.1
O1—Nd1—C16	70.42 (6)	C29—C30—H30A	119.1
O8—Nd1—C16	90.98 (6)	C25—C31—C32	114.9 (2)
N1—Nd1—C16	102.99 (7)	C25—C31—H31A	108.5
O4—Nd1—C8	93.67 (7)	C32—C31—H31A	108.5
O5—Nd1—C8	101.18 (7)	C25—C31—H31B	108.5
O10—Nd1—C8	90.43 (6)	C32—C31—H31B	108.5
O1W—Nd1—C8	100.61 (7)	H31A—C31—H31B	107.5
O2—Nd1—C8	25.51 (6)	O11—C32—O10	120.3 (2)
O7—Nd1—C8	151.62 (6)	O11—C32—C31	122.4 (2)
O1—Nd1—C8	25.81 (6)	O10—C32—C31	117.2 (2)
O8—Nd1—C8	156.75 (6)	O11—C32—Nd2	61.64 (12)
N1—Nd1—C8	77.14 (6)	O10—C32—Nd2	59.91 (11)
C16—Nd1—C8	95.80 (7)	C31—C32—Nd2	168.17 (18)
O4—Nd1—C24	91.45 (7)	C38—C33—C34	117.0 (2)
O5—Nd1—C24	87.01 (6)	C38—C33—C39	121.6 (2)
O10—Nd1—C24	91.85 (6)	C34—C33—C39	121.4 (2)
O1W—Nd1—C24	72.08 (7)	C35—C34—C33	122.1 (2)
O2—Nd1—C24	146.91 (6)	C35—C34—H34A	118.9
O7—Nd1—C24	24.89 (6)	C33—C34—H34A	118.9
O1—Nd1—C24	162.01 (7)	C34—C35—C36	120.3 (2)
O8—Nd1—C24	25.74 (6)	C34—C35—H35A	119.9
N1—Nd1—C24	97.85 (6)	C36—C35—H35A	119.9
C16—Nd1—C24	91.63 (7)	O15—C36—C37	123.1 (2)
C8—Nd1—C24	171.81 (7)	O15—C36—C35	118.1 (2)
O8—Nd2—O14	127.17 (5)	C37—C36—C35	118.8 (2)
O8—Nd2—O13	74.96 (5)	C36—C37—C38	120.2 (2)
O14—Nd2—O13	52.73 (5)	C36—C37—H37A	119.9
O8—Nd2—O2W	78.76 (6)	C38—C37—H37A	119.9
O14—Nd2—O2W	144.15 (6)	C37—C38—C33	121.5 (2)
O13—Nd2—O2W	147.27 (5)	C37—C38—H38A	119.3
O8—Nd2—O16	81.88 (5)	C33—C38—H38A	119.3
O14—Nd2—O16	126.32 (6)	C40—C39—C33	116.0 (2)
O13—Nd2—O16	118.12 (6)	C40—C39—H39A	108.3
O2W—Nd2—O16	76.34 (6)	C33—C39—H39A	108.3
O8—Nd2—O17	97.95 (6)	C40—C39—H39B	108.3
O14—Nd2—O17	78.44 (6)	C33—C39—H39B	108.3
O13—Nd2—O17	76.46 (6)	H39A—C39—H39B	107.4
O2W—Nd2—O17	126.81 (6)	O14—C40—O13	119.5 (2)
O16—Nd2—O17	50.94 (6)	O14—C40—C39	119.3 (2)
O8—Nd2—O10	67.63 (5)	O13—C40—C39	121.2 (2)
O14—Nd2—O10	90.57 (5)	O14—C40—Nd2	59.44 (12)
O13—Nd2—O10	75.76 (5)	O13—C40—Nd2	60.09 (12)
O2W—Nd2—O10	76.42 (6)	C39—C40—Nd2	177.45 (16)
O16—Nd2—O10	142.31 (5)	C46—C41—C42	117.2 (2)
O17—Nd2—O10	151.23 (5)	C46—C41—C47	120.7 (3)
O8—Nd2—O11	114.99 (5)	C42—C41—C47	122.1 (2)
O14—Nd2—O11	73.64 (6)	C43—C42—C41	122.0 (2)
O13—Nd2—O11	101.41 (6)	C43—C42—H42A	119.0
O2W—Nd2—O11	72.54 (6)	C41—C42—H42A	119.0
O16—Nd2—O11	140.16 (6)	C42—C43—C44	119.6 (3)
O17—Nd2—O11	145.48 (6)	C42—C43—H43A	120.2
O10—Nd2—O11	49.63 (5)	C44—C43—H43A	120.2
O8—Nd2—N3	155.81 (6)	O18—C44—C45	122.9 (3)
O14—Nd2—N3	75.75 (6)	O18—C44—C43	117.5 (3)
O13—Nd2—N3	125.68 (6)	C45—C44—C43	119.6 (3)
O2W—Nd2—N3	84.93 (6)	C44—C45—C46	120.3 (3)
O16—Nd2—N3	76.96 (6)	C44—C45—H45A	119.8
O17—Nd2—N3	77.67 (6)	C46—C45—H45A	119.8
O10—Nd2—N3	125.60 (5)	C45—C46—C41	121.2 (3)
O11—Nd2—N3	76.11 (5)	C45—C46—H46A	119.4
O8—Nd2—C40	101.29 (6)	C41—C46—H46A	119.4
O14—Nd2—C40	26.21 (6)	C41—C47—C48	112.0 (2)
O13—Nd2—C40	26.52 (6)	C41—C47—H47A	109.2
O2W—Nd2—C40	157.53 (7)	C48—C47—H47A	109.2
O16—Nd2—C40	126.09 (6)	C41—C47—H47B	109.2
O17—Nd2—C40	75.62 (6)	C48—C47—H47B	109.2
O10—Nd2—C40	82.81 (6)	H47A—C47—H47B	107.9
O11—Nd2—C40	87.53 (6)	O16—C48—O17	120.0 (2)
N3—Nd2—C40	100.59 (6)	O16—C48—C47	119.8 (2)
O8—Nd2—C48	92.04 (6)	O17—C48—C47	120.2 (2)
O14—Nd2—C48	101.96 (7)	O16—C48—Nd2	59.05 (13)
O13—Nd2—C48	99.02 (7)	O17—C48—Nd2	61.72 (12)
O2W—Nd2—C48	101.08 (7)	C47—C48—Nd2	170.41 (17)
O16—Nd2—C48	25.36 (6)	N1—C49—C50	123.1 (2)
O17—Nd2—C48	25.78 (6)	N1—C49—H49A	118.5
O10—Nd2—C48	159.65 (6)	C50—C49—H49A	118.5
O11—Nd2—C48	149.50 (6)	C51—C50—C49	119.7 (2)
N3—Nd2—C48	73.60 (6)	C51—C50—H50A	120.1
C40—Nd2—C48	101.38 (7)	C49—C50—H50A	120.1
O8—Nd2—C32	90.62 (6)	C50—C51—C52	117.0 (2)
O14—Nd2—C32	84.08 (6)	C50—C51—C56	121.4 (2)
O13—Nd2—C32	91.06 (6)	C52—C51—C56	121.5 (2)
O2W—Nd2—C32	69.93 (7)	C53—C52—C51	119.6 (2)
O16—Nd2—C32	146.25 (6)	C53—C52—H52A	120.2
O17—Nd2—C32	162.35 (6)	C51—C52—H52A	120.2
O10—Nd2—C32	25.13 (5)	N1—C53—C52	123.4 (2)
O11—Nd2—C32	24.82 (5)	N1—C53—H53A	118.3
N3—Nd2—C32	100.48 (6)	C52—C53—H53A	118.3
C40—Nd2—C32	87.62 (6)	N2—C54—C55	123.9 (3)
C48—Nd2—C32	169.92 (7)	N2—C54—H54A	118.0
C49—N1—C53	117.1 (2)	C55—C54—H54A	118.0
C49—N1—Nd1	124.12 (16)	C56—C55—C54	119.4 (3)
C53—N1—Nd1	118.35 (16)	C56—C55—H55A	120.3
C58—N2—C54	116.0 (3)	C54—C55—H55A	120.3
C59—N3—C63	116.8 (2)	C55—C56—C57	117.0 (3)
C59—N3—Nd2	121.34 (16)	C55—C56—C51	121.2 (3)
C63—N3—Nd2	121.34 (15)	C57—C56—C51	121.7 (3)
C64—N4—C68	115.9 (3)	C56—C57—C58	119.0 (3)
C2—C1—C6	117.6 (2)	C56—C57—H57A	120.5
C2—C1—C7	122.0 (2)	C58—C57—H57A	120.5
C6—C1—C7	120.4 (2)	N2—C58—C57	124.6 (3)
C1—C2—C3	121.5 (3)	N2—C58—H58A	117.7
C1—C2—H2A	119.3	C57—C58—H58A	117.7
C3—C2—H2A	119.3	N3—C59—C60	123.2 (2)
C4—C3—C2	119.7 (3)	N3—C59—H59A	118.4
C4—C3—H3A	120.1	C60—C59—H59A	118.4
C2—C3—H3A	120.1	C61—C60—C59	120.4 (2)
C3—C4—C5	119.8 (3)	C61—C60—H60A	119.8
C3—C4—O3	118.5 (3)	C59—C60—H60A	119.8
C5—C4—O3	121.7 (3)	C60—C61—C62	116.1 (2)
C4—C5—C6	120.2 (3)	C60—C61—C66	121.3 (2)
C4—C5—H5A	119.9	C62—C61—C66	122.5 (2)
C6—C5—H5A	119.9	C63—C62—C61	120.1 (2)
C5—C6—C1	121.2 (3)	C63—C62—H62A	119.9
C5—C6—H6A	119.4	C61—C62—H62A	119.9
C1—C6—H6A	119.4	N3—C63—C62	123.3 (2)
C8—C7—C1	111.7 (2)	N3—C63—H63A	118.4
C8—C7—H7A	109.3	C62—C63—H63A	118.4
C1—C7—H7A	109.3	N4—C64—C65	124.3 (3)
C8—C7—H7B	109.3	N4—C64—H64A	117.9
C1—C7—H7B	109.3	C65—C64—H64A	117.9
H7A—C7—H7B	107.9	C64—C65—C66	119.7 (3)
O2—C8—O1	120.1 (2)	C64—C65—H65A	120.2
O2—C8—C7	119.6 (2)	C66—C65—H65A	120.2
O1—C8—C7	120.3 (2)	C67—C66—C65	116.4 (3)
O2—C8—Nd1	58.40 (12)	C67—C66—C61	121.3 (3)
O1—C8—Nd1	62.51 (12)	C65—C66—C61	122.3 (3)
C7—C8—Nd1	168.90 (16)	C68—C67—C66	119.4 (3)
C10—C9—C14	117.0 (2)	C68—C67—H67A	120.3
C10—C9—C15	122.1 (2)	C66—C67—H67A	120.3
C14—C9—C15	120.9 (2)	N4—C68—C67	124.3 (4)
C9—C10—C11	122.2 (2)	N4—C68—H68A	117.9
C9—C10—H10A	118.9	C67—C68—H68A	117.9
C11—C10—H10A	118.9	Nd1—O1W—H1WA	118.6 (19)
C12—C11—C10	119.6 (2)	Nd1—O1W—H1WB	135.0 (19)
C12—C11—H11A	120.2	H1WA—O1W—H1WB	106 (2)
C10—C11—H11A	120.2	C8—O1—Nd1	91.69 (14)
C11—C12—O6	123.2 (2)	Nd2—O2W—H2WA	122.1 (19)
C11—C12—C13	119.3 (2)	Nd2—O2W—H2WB	130 (2)
O6—C12—C13	117.5 (2)	H2WA—O2W—H2WB	108 (2)
C14—C13—C12	120.3 (2)	C8—O2—Nd1	96.09 (15)
C14—C13—H13A	119.8	C4—O3—H3B	109.5
C12—C13—H13A	119.8	H3WA—O3W—H3WB	106 (2)
C13—C14—C9	121.5 (2)	C16—O4—Nd1	92.81 (14)
C13—C14—H14A	119.2	C16—O5—Nd1	92.54 (14)
C9—C14—H14A	119.2	C12—O6—H6B	109.5
C9—C15—C16	115.3 (2)	C24—O7—Nd1	96.79 (14)
C9—C15—H15A	108.4	C24—O8—Nd2	153.48 (15)
C16—C15—H15A	108.4	C24—O8—Nd1	90.94 (13)
C9—C15—H15B	108.4	Nd2—O8—Nd1	113.30 (6)
C16—C15—H15B	108.4	C20—O9—H9A	109.5
H15A—C15—H15B	107.5	C32—O10—Nd1	143.18 (14)
O4—C16—O5	120.9 (2)	C32—O10—Nd2	94.95 (13)
O4—C16—C15	118.9 (2)	Nd1—O10—Nd2	112.55 (6)
O5—C16—C15	120.1 (2)	C32—O11—Nd2	93.55 (13)
O4—C16—Nd1	60.79 (13)	C28—O12—H12A	109.5
O5—C16—Nd1	60.87 (12)	C40—O13—Nd2	93.39 (13)
C15—C16—Nd1	168.94 (19)	C40—O14—Nd2	94.34 (14)
C18—C17—C22	117.5 (3)	C36—O15—H15C	109.5
C18—C17—C23	121.0 (3)	C48—O16—Nd2	95.59 (15)
C22—C17—C23	121.4 (3)	C48—O17—Nd2	92.50 (14)
C17—C18—C19	121.9 (3)	C44—O18—H18B	109.5
C17—C18—H18A	119.0		
			
O4—Nd1—N1—C49	−148.29 (19)	O14—Nd2—C48—O16	−165.18 (13)
O5—Nd1—N1—C49	−145.32 (17)	O13—Nd2—C48—O16	141.20 (14)
O10—Nd1—N1—C49	9.3 (3)	O2W—Nd2—C48—O16	−12.84 (15)
O1W—Nd1—N1—C49	51.50 (18)	O17—Nd2—C48—O16	170.3 (2)
O2—Nd1—N1—C49	−25.26 (18)	O10—Nd2—C48—O16	67.9 (2)
O7—Nd1—N1—C49	130.97 (19)	O11—Nd2—C48—O16	−87.18 (19)
O1—Nd1—N1—C49	−75.10 (19)	N3—Nd2—C48—O16	−94.13 (14)
O8—Nd1—N1—C49	115.77 (18)	C40—Nd2—C48—O16	168.05 (14)
C16—Nd1—N1—C49	−144.43 (18)	C32—Nd2—C48—O16	−39.1 (4)
C8—Nd1—N1—C49	−51.35 (19)	O8—Nd2—C48—O17	−104.16 (15)
C24—Nd1—N1—C49	122.06 (19)	O14—Nd2—C48—O17	24.56 (15)
O4—Nd1—N1—C53	39.24 (18)	O13—Nd2—C48—O17	−29.06 (15)
O5—Nd1—N1—C53	42.2 (2)	O2W—Nd2—C48—O17	176.90 (14)
O10—Nd1—N1—C53	−163.16 (16)	O16—Nd2—C48—O17	−170.3 (2)
O1W—Nd1—N1—C53	−120.97 (19)	O10—Nd2—C48—O17	−102.3 (2)
O2—Nd1—N1—C53	162.27 (19)	O11—Nd2—C48—O17	102.57 (17)
O7—Nd1—N1—C53	−41.51 (18)	N3—Nd2—C48—O17	95.61 (15)
O1—Nd1—N1—C53	112.43 (18)	C40—Nd2—C48—O17	−2.21 (16)
O8—Nd1—N1—C53	−56.71 (19)	C32—Nd2—C48—O17	150.7 (3)
C16—Nd1—N1—C53	43.09 (19)	O8—Nd2—C48—C47	151.8 (12)
C8—Nd1—N1—C53	136.17 (19)	O14—Nd2—C48—C47	−79.5 (12)
C24—Nd1—N1—C53	−50.42 (18)	O13—Nd2—C48—C47	−133.1 (12)
O8—Nd2—N3—C59	0.7 (3)	O2W—Nd2—C48—C47	72.9 (12)
O14—Nd2—N3—C59	163.59 (19)	O16—Nd2—C48—C47	85.7 (12)
O13—Nd2—N3—C59	145.70 (17)	O17—Nd2—C48—C47	−104.0 (12)
O2W—Nd2—N3—C59	−46.85 (18)	O10—Nd2—C48—C47	153.7 (11)
O16—Nd2—N3—C59	30.27 (17)	O11—Nd2—C48—C47	−1.4 (13)
O17—Nd2—N3—C59	82.57 (18)	N3—Nd2—C48—C47	−8.4 (12)
O10—Nd2—N3—C59	−116.15 (17)	C40—Nd2—C48—C47	−106.2 (12)
O11—Nd2—N3—C59	−120.09 (18)	C32—Nd2—C48—C47	46.6 (14)
C40—Nd2—N3—C59	155.15 (18)	C53—N1—C49—C50	1.8 (4)
C48—Nd2—N3—C59	56.28 (18)	Nd1—N1—C49—C50	−170.76 (18)
C32—Nd2—N3—C59	−115.33 (18)	N1—C49—C50—C51	0.2 (4)
O8—Nd2—N3—C63	171.95 (15)	C49—C50—C51—C52	−1.8 (4)
O14—Nd2—N3—C63	−25.13 (17)	C49—C50—C51—C56	176.3 (2)
O13—Nd2—N3—C63	−43.03 (19)	C50—C51—C52—C53	1.4 (4)
O2W—Nd2—N3—C63	124.42 (18)	C56—C51—C52—C53	−176.6 (2)
O16—Nd2—N3—C63	−158.45 (18)	C49—N1—C53—C52	−2.2 (4)
O17—Nd2—N3—C63	−106.15 (18)	Nd1—N1—C53—C52	170.8 (2)
O10—Nd2—N3—C63	55.12 (19)	C51—C52—C53—N1	0.6 (4)
O11—Nd2—N3—C63	51.18 (17)	C58—N2—C54—C55	0.9 (5)
C40—Nd2—N3—C63	−33.58 (18)	N2—C54—C55—C56	0.2 (5)
C48—Nd2—N3—C63	−132.45 (19)	C54—C55—C56—C57	−1.5 (4)
C32—Nd2—N3—C63	55.94 (18)	C54—C55—C56—C51	176.0 (3)
C6—C1—C2—C3	−1.7 (4)	C50—C51—C56—C55	−148.1 (3)
C7—C1—C2—C3	−179.1 (2)	C52—C51—C56—C55	29.8 (4)
C1—C2—C3—C4	1.2 (4)	C50—C51—C56—C57	29.3 (4)
C2—C3—C4—C5	0.9 (4)	C52—C51—C56—C57	−152.7 (3)
C2—C3—C4—O3	−179.6 (3)	C55—C56—C57—C58	1.8 (4)
C3—C4—C5—C6	−2.4 (4)	C51—C56—C57—C58	−175.7 (3)
O3—C4—C5—C6	178.2 (3)	C54—N2—C58—C57	−0.6 (5)
C4—C5—C6—C1	1.8 (4)	C56—C57—C58—N2	−0.8 (5)
C2—C1—C6—C5	0.2 (4)	C63—N3—C59—C60	−2.0 (4)
C7—C1—C6—C5	177.7 (2)	Nd2—N3—C59—C60	169.66 (18)
C2—C1—C7—C8	−49.4 (3)	N3—C59—C60—C61	−1.0 (4)
C6—C1—C7—C8	133.2 (3)	C59—C60—C61—C62	3.0 (4)
C1—C7—C8—O2	−77.0 (3)	C59—C60—C61—C66	−174.1 (2)
C1—C7—C8—O1	101.1 (3)	C60—C61—C62—C63	−2.0 (4)
C1—C7—C8—Nd1	−0.3 (11)	C66—C61—C62—C63	175.0 (2)
O4—Nd1—C8—O2	153.70 (14)	C59—N3—C63—C62	3.0 (4)
O5—Nd1—C8—O2	−153.55 (13)	Nd2—N3—C63—C62	−168.67 (18)
O10—Nd1—C8—O2	−81.01 (14)	C61—C62—C63—N3	−1.0 (4)
O1W—Nd1—C8—O2	−1.15 (14)	C68—N4—C64—C65	0.5 (5)
O7—Nd1—C8—O2	82.8 (2)	N4—C64—C65—C66	1.3 (5)
O1—Nd1—C8—O2	−169.5 (2)	C64—C65—C66—C67	−1.6 (4)
O8—Nd1—C8—O2	−73.7 (2)	C64—C65—C66—C61	175.0 (3)
N1—Nd1—C8—O2	78.06 (14)	C60—C61—C66—C67	159.8 (3)
C16—Nd1—C8—O2	−179.90 (14)	C62—C61—C66—C67	−17.1 (4)
C24—Nd1—C8—O2	25.1 (5)	C60—C61—C66—C65	−16.6 (4)
O4—Nd1—C8—O1	−36.83 (14)	C62—C61—C66—C65	166.5 (3)
O5—Nd1—C8—O1	15.93 (15)	C65—C66—C67—C68	0.3 (4)
O10—Nd1—C8—O1	88.47 (14)	C61—C66—C67—C68	−176.3 (3)
O1W—Nd1—C8—O1	168.33 (14)	C64—N4—C68—C67	−1.9 (5)
O2—Nd1—C8—O1	169.5 (2)	C66—C67—C68—N4	1.6 (5)
O7—Nd1—C8—O1	−107.74 (18)	O2—C8—O1—Nd1	10.4 (2)
O8—Nd1—C8—O1	95.8 (2)	C7—C8—O1—Nd1	−167.7 (2)
N1—Nd1—C8—O1	−112.46 (15)	O4—Nd1—O1—C8	141.32 (15)
C16—Nd1—C8—O1	−10.42 (15)	O5—Nd1—O1—C8	−163.92 (15)
C24—Nd1—C8—O1	−165.4 (4)	O10—Nd1—O1—C8	−91.56 (14)
O4—Nd1—C8—C7	70.6 (10)	O1W—Nd1—O1—C8	−14.19 (17)
O5—Nd1—C8—C7	123.3 (10)	O2—Nd1—O1—C8	−5.80 (13)
O10—Nd1—C8—C7	−164.1 (10)	O7—Nd1—O1—C8	119.17 (16)
O1W—Nd1—C8—C7	−84.3 (10)	O8—Nd1—O1—C8	−132.82 (14)
O2—Nd1—C8—C7	−83.1 (10)	N1—Nd1—O1—C8	64.37 (14)
O7—Nd1—C8—C7	−0.3 (11)	C16—Nd1—O1—C8	168.99 (16)
O1—Nd1—C8—C7	107.4 (10)	C24—Nd1—O1—C8	173.32 (19)
O8—Nd1—C8—C7	−156.8 (9)	O1—C8—O2—Nd1	−10.8 (2)
N1—Nd1—C8—C7	−5.0 (10)	C7—C8—O2—Nd1	167.29 (19)
C16—Nd1—C8—C7	97.0 (10)	O4—Nd1—O2—C8	−29.55 (15)
C24—Nd1—C8—C7	−58.0 (12)	O5—Nd1—O2—C8	31.64 (16)
C14—C9—C10—C11	1.2 (4)	O10—Nd1—O2—C8	98.31 (14)
C15—C9—C10—C11	−178.8 (3)	O1W—Nd1—O2—C8	178.83 (15)
C9—C10—C11—C12	−1.2 (4)	O7—Nd1—O2—C8	−132.55 (14)
C10—C11—C12—O6	179.3 (3)	O1—Nd1—O2—C8	5.87 (13)
C10—C11—C12—C13	0.7 (4)	O8—Nd1—O2—C8	142.62 (13)
C11—C12—C13—C14	−0.3 (4)	N1—Nd1—O2—C8	−95.16 (14)
O6—C12—C13—C14	−179.0 (3)	C16—Nd1—O2—C8	0.12 (16)
C12—C13—C14—C9	0.3 (4)	C24—Nd1—O2—C8	−173.64 (14)
C10—C9—C14—C13	−0.7 (4)	O5—C16—O4—Nd1	10.2 (2)
C15—C9—C14—C13	179.2 (3)	C15—C16—O4—Nd1	−167.3 (2)
C10—C9—C15—C16	−100.4 (3)	O5—Nd1—O4—C16	−5.61 (14)
C14—C9—C15—C16	79.7 (3)	O10—Nd1—O4—C16	2.53 (17)
C9—C15—C16—O4	172.4 (2)	O1W—Nd1—O4—C16	−144.41 (15)
C9—C15—C16—O5	−5.1 (4)	O2—Nd1—O4—C16	107.93 (15)
C9—C15—C16—Nd1	86.9 (9)	O7—Nd1—O4—C16	−111.71 (15)
O5—Nd1—C16—O4	170.0 (2)	O1—Nd1—O4—C16	79.82 (15)
O10—Nd1—C16—O4	−177.91 (14)	O8—Nd1—O4—C16	−67.08 (15)
O1W—Nd1—C16—O4	97.8 (3)	N1—Nd1—O4—C16	171.52 (16)
O2—Nd1—C16—O4	−86.63 (16)	C8—Nd1—O4—C16	95.65 (15)
O7—Nd1—C16—O4	65.29 (15)	C24—Nd1—O4—C16	−90.75 (15)
O1—Nd1—C16—O4	−91.37 (15)	O4—C16—O5—Nd1	−10.2 (2)
O8—Nd1—C16—O4	115.71 (15)	C15—C16—O5—Nd1	167.3 (2)
N1—Nd1—C16—O4	−8.47 (16)	O4—Nd1—O5—C16	5.57 (14)
C8—Nd1—C16—O4	−86.57 (15)	O10—Nd1—O5—C16	−167.47 (15)
C24—Nd1—C16—O4	89.97 (15)	O1W—Nd1—O5—C16	151.98 (14)
O4—Nd1—C16—O5	−170.0 (2)	O2—Nd1—O5—C16	−93.83 (15)
O10—Nd1—C16—O5	12.10 (15)	O7—Nd1—O5—C16	76.08 (14)
O1W—Nd1—C16—O5	−72.2 (3)	O1—Nd1—O5—C16	−73.46 (14)
O2—Nd1—C16—O5	103.39 (14)	O8—Nd1—O5—C16	123.12 (15)
O7—Nd1—C16—O5	−104.70 (14)	N1—Nd1—O5—C16	1.95 (17)
O1—Nd1—C16—O5	98.64 (15)	C8—Nd1—O5—C16	−80.52 (15)
O8—Nd1—C16—O5	−54.28 (14)	C24—Nd1—O5—C16	99.67 (15)
N1—Nd1—C16—O5	−178.46 (14)	O8—C24—O7—Nd1	−8.9 (3)
C8—Nd1—C16—O5	103.44 (14)	C23—C24—O7—Nd1	170.0 (2)
C24—Nd1—C16—O5	−80.02 (14)	O4—Nd1—O7—C24	121.84 (16)
O4—Nd1—C16—C15	91.7 (9)	O5—Nd1—O7—C24	71.67 (16)
O5—Nd1—C16—C15	−98.3 (9)	O10—Nd1—O7—C24	−1.60 (17)
O10—Nd1—C16—C15	−86.2 (9)	O1W—Nd1—O7—C24	−73.62 (16)
O1W—Nd1—C16—C15	−170.5 (8)	O2—Nd1—O7—C24	−121.51 (15)
O2—Nd1—C16—C15	5.1 (9)	O1—Nd1—O7—C24	143.50 (15)
O7—Nd1—C16—C15	157.0 (9)	O8—Nd1—O7—C24	4.81 (14)
O1—Nd1—C16—C15	0.3 (9)	N1—Nd1—O7—C24	−158.61 (17)
O8—Nd1—C16—C15	−152.6 (9)	C16—Nd1—O7—C24	97.43 (16)
N1—Nd1—C16—C15	83.2 (9)	C8—Nd1—O7—C24	−163.38 (16)
C8—Nd1—C16—C15	5.1 (9)	O7—C24—O8—Nd2	165.3 (2)
C24—Nd1—C16—C15	−178.3 (9)	C23—C24—O8—Nd2	−13.7 (5)
C22—C17—C18—C19	0.1 (4)	Nd1—C24—O8—Nd2	156.8 (4)
C23—C17—C18—C19	178.7 (2)	O7—C24—O8—Nd1	8.5 (2)
C17—C18—C19—C20	0.5 (4)	C23—C24—O8—Nd1	−170.5 (2)
C18—C19—C20—O9	179.8 (2)	O14—Nd2—O8—C24	−82.1 (4)
C18—C19—C20—C21	−1.0 (4)	O13—Nd2—O8—C24	−74.2 (4)
O9—C20—C21—C22	−179.7 (2)	O2W—Nd2—O8—C24	125.5 (4)
C19—C20—C21—C22	1.1 (4)	O16—Nd2—O8—C24	47.9 (4)
C20—C21—C22—C17	−0.5 (4)	O17—Nd2—O8—C24	−0.6 (4)
C18—C17—C22—C21	0.0 (4)	O10—Nd2—O8—C24	−154.7 (4)
C23—C17—C22—C21	−178.6 (2)	O11—Nd2—O8—C24	−170.0 (3)
C18—C17—C23—C24	−80.7 (3)	N3—Nd2—O8—C24	77.0 (4)
C22—C17—C23—C24	97.9 (3)	C40—Nd2—O8—C24	−77.4 (4)
C17—C23—C24—O7	−45.7 (4)	C48—Nd2—O8—C24	24.6 (4)
C17—C23—C24—O8	133.3 (3)	C32—Nd2—O8—C24	−165.1 (4)
C17—C23—C24—Nd1	29.4 (14)	O14—Nd2—O8—Nd1	72.48 (9)
O4—Nd1—C24—O7	−56.16 (16)	O13—Nd2—O8—Nd1	80.37 (7)
O5—Nd1—C24—O7	−108.76 (16)	O2W—Nd2—O8—Nd1	−79.94 (7)
O10—Nd1—C24—O7	178.57 (16)	O16—Nd2—O8—Nd1	−157.53 (7)
O1W—Nd1—C24—O7	99.75 (16)	O17—Nd2—O8—Nd1	153.97 (6)
O2—Nd1—C24—O7	92.10 (19)	O10—Nd2—O8—Nd1	−0.14 (5)
O1—Nd1—C24—O7	−86.7 (3)	O11—Nd2—O8—Nd1	−15.42 (8)
O8—Nd1—C24—O7	−171.4 (2)	N3—Nd2—O8—Nd1	−128.46 (12)
N1—Nd1—C24—O7	20.79 (16)	C40—Nd2—O8—Nd1	77.14 (7)
C16—Nd1—C24—O7	−82.57 (16)	C48—Nd2—O8—Nd1	179.17 (7)
C8—Nd1—C24—O7	72.5 (5)	C32—Nd2—O8—Nd1	−10.56 (7)
O4—Nd1—C24—O8	115.28 (14)	O4—Nd1—O8—C24	−67.55 (14)
O5—Nd1—C24—O8	62.67 (14)	O5—Nd1—O8—C24	−113.75 (15)
O10—Nd1—C24—O8	−9.99 (14)	O10—Nd1—O8—C24	169.10 (15)
O1W—Nd1—C24—O8	−88.82 (14)	O1W—Nd1—O8—C24	83.05 (14)
O2—Nd1—C24—O8	−96.46 (16)	O2—Nd1—O8—C24	119.62 (15)
O7—Nd1—C24—O8	171.4 (2)	O7—Nd1—O8—C24	−4.66 (14)
O1—Nd1—C24—O8	84.8 (3)	O1—Nd1—O8—C24	−144.94 (15)
N1—Nd1—C24—O8	−167.78 (13)	N1—Nd1—O8—C24	14.47 (16)
C16—Nd1—C24—O8	88.87 (14)	C16—Nd1—O8—C24	−91.73 (15)
C8—Nd1—C24—O8	−116.0 (4)	C8—Nd1—O8—C24	161.08 (17)
O4—Nd1—C24—C23	−136.4 (12)	O4—Nd1—O8—Nd2	123.50 (7)
O5—Nd1—C24—C23	171.0 (12)	O5—Nd1—O8—Nd2	77.30 (7)
O10—Nd1—C24—C23	98.4 (12)	O10—Nd1—O8—Nd2	0.15 (5)
O1W—Nd1—C24—C23	19.5 (12)	O1W—Nd1—O8—Nd2	−85.90 (7)
O2—Nd1—C24—C23	11.9 (13)	O2—Nd1—O8—Nd2	−49.33 (11)
O7—Nd1—C24—C23	−80.2 (12)	O7—Nd1—O8—Nd2	−173.61 (10)
O1—Nd1—C24—C23	−166.9 (11)	O1—Nd1—O8—Nd2	46.11 (13)
O8—Nd1—C24—C23	108.4 (12)	N1—Nd1—O8—Nd2	−154.48 (7)
N1—Nd1—C24—C23	−59.4 (12)	C16—Nd1—O8—Nd2	99.32 (7)
C16—Nd1—C24—C23	−162.8 (12)	C8—Nd1—O8—Nd2	−7.87 (19)
C8—Nd1—C24—C23	−7.7 (15)	C24—Nd1—O8—Nd2	−168.95 (17)
C30—C25—C26—C27	−0.2 (4)	O11—C32—O10—Nd1	−152.18 (18)
C31—C25—C26—C27	178.1 (2)	C31—C32—O10—Nd1	27.6 (4)
C25—C26—C27—C28	−0.7 (4)	Nd2—C32—O10—Nd1	−139.3 (2)
C26—C27—C28—O12	−179.1 (2)	O11—C32—O10—Nd2	−12.9 (2)
C26—C27—C28—C29	0.9 (4)	C31—C32—O10—Nd2	166.9 (2)
O12—C28—C29—C30	179.8 (2)	O4—Nd1—O10—C32	46.8 (3)
C27—C28—C29—C30	−0.1 (4)	O5—Nd1—O10—C32	53.6 (2)
C26—C25—C30—C29	1.0 (4)	O1W—Nd1—O10—C32	−148.7 (3)
C31—C25—C30—C29	−177.3 (2)	O2—Nd1—O10—C32	−73.2 (2)
C28—C29—C30—C25	−0.8 (4)	O7—Nd1—O10—C32	140.5 (2)
C30—C25—C31—C32	75.4 (3)	O1—Nd1—O10—C32	−22.2 (3)
C26—C25—C31—C32	−102.8 (3)	O8—Nd1—O10—C32	135.2 (3)
C25—C31—C32—O11	11.7 (4)	N1—Nd1—O10—C32	−106.2 (3)
C25—C31—C32—O10	−168.1 (2)	C16—Nd1—O10—C32	47.9 (3)
C25—C31—C32—Nd2	−95.1 (8)	C8—Nd1—O10—C32	−48.0 (3)
O8—Nd2—C32—O11	−169.46 (14)	C24—Nd1—O10—C32	139.9 (2)
O14—Nd2—C32—O11	63.22 (14)	O4—Nd1—O10—Nd2	−88.51 (8)
O13—Nd2—C32—O11	115.57 (14)	O5—Nd1—O10—Nd2	−81.73 (7)
O2W—Nd2—C32—O11	−91.67 (14)	O1W—Nd1—O10—Nd2	76.00 (7)
O16—Nd2—C32—O11	−93.24 (16)	O2—Nd1—O10—Nd2	151.48 (7)
O17—Nd2—C32—O11	71.2 (3)	O7—Nd1—O10—Nd2	5.24 (9)
O10—Nd2—C32—O11	167.4 (2)	O1—Nd1—O10—Nd2	−157.50 (7)
N3—Nd2—C32—O11	−11.06 (15)	O8—Nd1—O10—Nd2	−0.14 (5)
C40—Nd2—C32—O11	89.27 (14)	N1—Nd1—O10—Nd2	118.49 (13)
C48—Nd2—C32—O11	−64.2 (4)	C16—Nd1—O10—Nd2	−87.37 (7)
O8—Nd2—C32—O10	23.18 (14)	C8—Nd1—O10—Nd2	176.70 (7)
O14—Nd2—C32—O10	−104.15 (13)	C24—Nd1—O10—Nd2	4.57 (7)
O13—Nd2—C32—O10	−51.80 (13)	O8—Nd2—O10—C32	−154.81 (15)
O2W—Nd2—C32—O10	100.96 (14)	O14—Nd2—O10—C32	74.70 (14)
O16—Nd2—C32—O10	99.40 (15)	O13—Nd2—O10—C32	125.85 (14)
O17—Nd2—C32—O10	−96.2 (2)	O2W—Nd2—O10—C32	−71.56 (14)
O11—Nd2—C32—O10	−167.4 (2)	O16—Nd2—O10—C32	−116.31 (14)
N3—Nd2—C32—O10	−178.43 (13)	O17—Nd2—O10—C32	141.23 (15)
C40—Nd2—C32—O10	−78.10 (14)	O11—Nd2—O10—C32	6.92 (13)
C48—Nd2—C32—O10	128.5 (4)	N3—Nd2—O10—C32	1.90 (15)
O8—Nd2—C32—C31	−56.1 (8)	C40—Nd2—O10—C32	99.80 (14)
O14—Nd2—C32—C31	176.6 (8)	C48—Nd2—O10—C32	−156.80 (19)
O13—Nd2—C32—C31	−131.1 (8)	O8—Nd2—O10—Nd1	0.15 (5)
O2W—Nd2—C32—C31	21.7 (8)	O14—Nd2—O10—Nd1	−130.34 (6)
O16—Nd2—C32—C31	20.1 (8)	O13—Nd2—O10—Nd1	−79.19 (7)
O17—Nd2—C32—C31	−175.5 (7)	O2W—Nd2—O10—Nd1	83.41 (7)
O10—Nd2—C32—C31	−79.3 (8)	O16—Nd2—O10—Nd1	38.65 (11)
O11—Nd2—C32—C31	113.4 (8)	O17—Nd2—O10—Nd1	−63.81 (14)
N3—Nd2—C32—C31	102.3 (8)	O11—Nd2—O10—Nd1	161.89 (10)
C40—Nd2—C32—C31	−157.4 (8)	N3—Nd2—O10—Nd1	156.86 (6)
C48—Nd2—C32—C31	49.2 (10)	C40—Nd2—O10—Nd1	−105.23 (7)
C38—C33—C34—C35	−0.8 (4)	C48—Nd2—O10—Nd1	−1.8 (2)
C39—C33—C34—C35	179.0 (2)	C32—Nd2—O10—Nd1	154.96 (16)
C33—C34—C35—C36	0.4 (4)	O10—C32—O11—Nd2	12.7 (2)
C34—C35—C36—O15	−179.7 (2)	C31—C32—O11—Nd2	−167.1 (2)
C34—C35—C36—C37	0.0 (4)	O8—Nd2—O11—C32	11.64 (15)
O15—C36—C37—C38	179.6 (2)	O14—Nd2—O11—C32	−112.26 (15)
C35—C36—C37—C38	−0.1 (4)	O13—Nd2—O11—C32	−66.94 (14)
C36—C37—C38—C33	−0.3 (4)	O2W—Nd2—O11—C32	79.80 (14)
C34—C33—C38—C37	0.8 (4)	O16—Nd2—O11—C32	120.02 (14)
C39—C33—C38—C37	−179.1 (2)	O17—Nd2—O11—C32	−149.58 (14)
C38—C33—C39—C40	−114.6 (3)	O10—Nd2—O11—C32	−7.00 (13)
C34—C33—C39—C40	65.6 (3)	N3—Nd2—O11—C32	168.79 (15)
C33—C39—C40—O14	174.1 (2)	C40—Nd2—O11—C32	−89.67 (14)
C33—C39—C40—O13	−7.1 (3)	C48—Nd2—O11—C32	161.92 (16)
C33—C39—C40—Nd2	−127 (4)	O14—C40—O13—Nd2	1.4 (2)
O8—Nd2—C40—O14	−171.57 (13)	C39—C40—O13—Nd2	−177.45 (19)
O13—Nd2—C40—O14	−178.6 (2)	O8—Nd2—O13—C40	−172.89 (14)
O2W—Nd2—C40—O14	−83.7 (2)	O14—Nd2—O13—C40	−0.79 (13)
O16—Nd2—C40—O14	100.30 (15)	O2W—Nd2—O13—C40	−135.21 (15)
O17—Nd2—C40—O14	93.01 (14)	O16—Nd2—O13—C40	115.13 (14)
O10—Nd2—C40—O14	−106.18 (14)	O17—Nd2—O13—C40	84.87 (14)
O11—Nd2—C40—O14	−56.58 (14)	O10—Nd2—O13—C40	−102.68 (14)
N3—Nd2—C40—O14	18.80 (15)	O11—Nd2—O13—C40	−59.81 (14)
C48—Nd2—C40—O14	94.00 (14)	N3—Nd2—O13—C40	21.18 (16)
C32—Nd2—C40—O14	−81.42 (14)	C48—Nd2—O13—C40	97.42 (14)
O8—Nd2—C40—O13	7.00 (14)	C32—Nd2—O13—C40	−82.53 (14)
O14—Nd2—C40—O13	178.6 (2)	O13—C40—O14—Nd2	−1.4 (2)
O2W—Nd2—C40—O13	94.9 (2)	C39—C40—O14—Nd2	177.46 (19)
O16—Nd2—C40—O13	−81.12 (15)	O8—Nd2—O14—C40	10.39 (17)
O17—Nd2—C40—O13	−88.42 (14)	O13—Nd2—O14—C40	0.80 (13)
O10—Nd2—C40—O13	72.39 (13)	O2W—Nd2—O14—C40	139.55 (14)
O11—Nd2—C40—O13	122.00 (14)	O16—Nd2—O14—C40	−99.32 (14)
N3—Nd2—C40—O13	−162.63 (13)	O17—Nd2—O14—C40	−80.88 (14)
C48—Nd2—C40—O13	−87.43 (14)	O10—Nd2—O14—C40	72.34 (14)
C32—Nd2—C40—O13	97.16 (14)	O11—Nd2—O14—C40	119.65 (14)
O8—Nd2—C40—C39	128 (4)	N3—Nd2—O14—C40	−160.92 (15)
O14—Nd2—C40—C39	−60 (4)	C48—Nd2—O14—C40	−91.51 (14)
O13—Nd2—C40—C39	121 (4)	C32—Nd2—O14—C40	96.66 (14)
O2W—Nd2—C40—C39	−144 (4)	O17—C48—O16—Nd2	9.9 (2)
O16—Nd2—C40—C39	40 (4)	C47—C48—O16—Nd2	−168.96 (19)
O17—Nd2—C40—C39	33 (4)	O8—Nd2—O16—C48	−112.64 (14)
O10—Nd2—C40—C39	−166 (4)	O14—Nd2—O16—C48	18.09 (16)
O11—Nd2—C40—C39	−117 (4)	O13—Nd2—O16—C48	−44.57 (15)
N3—Nd2—C40—C39	−42 (4)	O2W—Nd2—O16—C48	167.03 (15)
C48—Nd2—C40—C39	34 (4)	O17—Nd2—O16—C48	−5.44 (13)
C32—Nd2—C40—C39	−142 (4)	O10—Nd2—O16—C48	−148.19 (13)
C46—C41—C42—C43	−0.8 (4)	O11—Nd2—O16—C48	127.69 (14)
C47—C41—C42—C43	177.0 (2)	N3—Nd2—O16—C48	79.15 (14)
C41—C42—C43—C44	−0.5 (4)	C40—Nd2—O16—C48	−14.55 (17)
C42—C43—C44—O18	−178.5 (2)	C32—Nd2—O16—C48	168.54 (13)
C42—C43—C44—C45	1.5 (4)	O16—C48—O17—Nd2	−9.7 (2)
O18—C44—C45—C46	178.7 (3)	C47—C48—O17—Nd2	169.2 (2)
C43—C44—C45—C46	−1.3 (4)	O8—Nd2—O17—C48	78.08 (15)
C44—C45—C46—C41	0.0 (4)	O14—Nd2—O17—C48	−155.48 (15)
C42—C41—C46—C45	1.0 (4)	O13—Nd2—O17—C48	150.43 (15)
C47—C41—C46—C45	−176.8 (2)	O2W—Nd2—O17—C48	−3.80 (17)
C46—C41—C47—C48	107.6 (3)	O16—Nd2—O17—C48	5.36 (13)
C42—C41—C47—C48	−70.1 (3)	O10—Nd2—O17—C48	135.10 (14)
C41—C47—C48—O16	57.1 (3)	O11—Nd2—O17—C48	−119.06 (15)
C41—C47—C48—O17	−121.8 (3)	N3—Nd2—O17—C48	−77.75 (15)
C41—C47—C48—Nd2	−23.1 (13)	C40—Nd2—O17—C48	177.76 (16)
O8—Nd2—C48—O16	66.10 (14)	C32—Nd2—O17—C48	−163.57 (18)

### Hydrogen-bond geometry (Å, °)

**Table:**

*D*—H···*A*	*D*—H	H···*A*	*D*···*A*	*D*—H···*A*
O3—H3B···O12^i^	0.82	1.94	2.751 (3)	170
O6—H6B···O3W^ii^	0.82	1.86	2.647 (3)	160
O9—H9A···O17^iii^	0.82	1.86	2.676 (3)	173
O12—H12A···O11^iv^	0.82	1.94	2.746 (2)	167
O15—H15C···O6^v^	0.82	1.91	2.725 (3)	173
O18—H18B···O9^ii^	0.82	1.95	2.763 (3)	173
O2W—H2WA···O5	0.82 (2)	2.02 (2)	2.772 (2)	153 (3)
O2W—H2WB···N2^ii^	0.81 (2)	2.04 (2)	2.840 (3)	170 (3)
O3W—H3WB···O3	0.82 (2)	2.02 (2)	2.811 (3)	162 (3)
O1W—H1WA···O13	0.81 (2)	1.98 (2)	2.764 (2)	161 (3)
O1W—H1WB···N4^i^	0.82 (2)	1.99 (2)	2.775 (3)	161 (3)
O3W—H3WA···O1^vi^	0.82 (2)	1.95 (2)	2.770 (3)	175 (4)
C6—H6A···O2^vii^	0.93	2.49	3.283 (3)	144
C31—H31A···O1	0.97	2.58	3.338 (3)	135
C49—H49A···O2	0.93	2.42	3.038 (3)	124
C59—H59A···O16	0.93	2.57	3.191 (3)	125
C63—H63A···O14	0.93	2.45	3.095 (3)	126

Symmetry codes: (i) *x*, *y*+1, *z*; (ii) *x*, *y*−1, *z*; (iii) −*x*, −*y*+1, −*z*; (iv) −*x*, −*y*, −*z*+1; (v) *x*−1, *y*+1, *z*; (vi) −*x*+1, −*y*+1, −*z*+1; (vii) −*x*, −*y*+1, −*z*+1.
